# Commentary: Donor Site Outcomes Following Autologous Breast Reconstruction with DIEP Flap: A Retrospective and Prospective Study in a Single Institution

**DOI:** 10.1177/22925503241292357

**Published:** 2024-10-23

**Authors:** Hillary Nepon, Tyler Safran, Joshua Vorstenbosch

**Affiliations:** 1 Division of Plastic and Reconstructive Surgery, 5620 McGill University, Montreal, Quebec, Canada

The deep inferior epigastric perforator (DIEP) flap has become the gold standard of autologous-based breast reconstruction. While the DIEP flap demonstrates favorable donor site complications compared to other abdominal-based flap breast reconstruction options,^1,2^ rates of DIEP donor site complications remain variable^3^ within the literature, and further research is required. In this study by Fan et al,^4^ the authors investigate the institutional rates of DIEP donor site complications using both retrospective and prospective modalities.

The retrospective component of the study reviewed whether abdominal donor site complications occurred in 117 patients following DIEP breast reconstruction from 2015 to 2021. They found that 41% of DIEP patients experienced at least 1 donor site complication with a statistically higher rate among bilateral versus unilateral reconstructions which is similar to other studies.^3,5,6^ Their finding that bilateral versus unilateral DIEP reconstruction also corresponds with the previous literature demonstrating that operative time varies directly with donor site morbidity.^7^ The prospective study component involved measuring donor site complications in 17 patients undergoing DIEP breast reconstruction from 2022 to 2023 and assessment of fluid collections and abdominal tissue edema with point-of-care ultrasound (POCUS). Similarly to the retrospective rate, 42.8% of prospective patients experienced a donor site complication. Their novel use of POCUS in the context of DIEP donor site morbidity further revealed that 23.5% of patients had a detectible fluid collection but a statistically stable change in mean abdominal tissue edema at −1.8% after 2–6 weeks.

Strengths of this study include prospective data collection, and the use of POCUS to investigate donor site edema and fluid collections which may remain clinically silent, highlighting the potential benefit of using POCUS within the postoperative clinic setting. The authors proposed options to improve the rate of collections in their study including progressive tension sutures and negative pressure wound therapy and we additionally suggest subcarpal defatting and topical tranexamic acid which have shown good results.^8^ The main weakness of the study was the retrospective nature of the chart review, and the authors highlighted how difficult it can be to ascertain details from written charts. Furthermore, their study was limited by a small sample size which may have been underpowered to detect statistical differences among their risk factor groups. It would be interesting to delve deeper into other risk factors present among their patient population including the rate of diabetes, preoperative nutritional status, and chemotherapy history. Overall, the study demonstrates the importance of evaluation of DIEP breast reconstruction donor site morbidity, and the authors importantly highlight how continuous quality improvement loops can evolve our practice patterns to improve surgical outcomes.

We congratulate the authors of this article on striving for transparent quality improvement by investigating and sharing their rates of abdominal donor site complications following DIEP breast reconstruction. This manuscript highlights the prevalence of donor site complications and the potential for optimizing surgical outcomes following DIEP flap breast reconstruction. We look forward to reading more of the work from this group in the future.
